# Predictors of Failed Intrauterine Balloon Tamponade in the Management of Severe Postpartum Hemorrhage

**DOI:** 10.3389/fmed.2021.656422

**Published:** 2021-07-15

**Authors:** Congcong Liu, Jinsong Gao, Juntao Liu, Xietong Wang, Jing He, Jingxia Sun, Xiaowei Liu, Shixiu Liao

**Affiliations:** ^1^Department of Obstetrics and Gynecology, Peking Union Medical College Hospital, Peking Union Medical College, Chinese Academy of Medical Sciences, Beijing, China; ^2^Department of Obstetrics and Gynecology, Shandong Provincial Hospital Affiliated to Shandong University, Jinan, China; ^3^Department of Obstetrics and Gynecology, Women's Hospital, School of Medicine, Zhejiang University, Hangzhou, China; ^4^Department of Obstetrics and Gynecology, The First Clinical Hospital Affiliated to Harbin Medical University, Harbin, China; ^5^Department of Obstetrics and Gynecology, Beijing Obstetrics and Gynecology Hospital, Capital Medical University, Beijing, China; ^6^Department of Obstetrics and Gynecology, Henan Provincial People's Hospital, Zhengzhou, China

**Keywords:** intrauterine balloon tamponade, postpartum hemorrhage, hysterectomy, placenta accreta spectrum, multiple gestation

## Abstract

To identify the factors predicting intrauterine balloon tamponade (IUBT) failure for severe postpartum hemorrhage (PPH) after delivery, we conducted a retrospective cohort study of women who underwent IUBT for severe PPH after delivery from October 1, 2016 until September 30, 2017. The failure of IUBT was defined as the need of additional surgical procedures or uterine embolization. A total of 99,650 deliveries occurred during the study period. Among the patients, 106 cases of severe PPH were managed with IUBT, and the global success rate was 70.8% (75/106). Least absolute shrinkage and selection operator (LASSO) regression was performed to select the potential risk factors predicting IUBT failure. The associated risk factors—obesity, multiple gestation, cesarean delivery, estimated blood loss (EBL), and placenta accreta spectrum (PAS)—were included in multivariate logistic models. Ultimately, these models identified multiple gestation, EBL, and PAS as independent risk factors for IUBT failure. In conclusion, IUBT is an effective method for severe PPH. The presence of factors affecting IUBT failure should be recognized early, and other modalities of management should be anticipated.

## Introduction

Postpartum hemorrhage (PPH) is still a major cause of maternal mortality worldwide (1). The universal two-child policy in China has been associated with a higher proportion of elderly pregnant women, and the incidence of PPH has significantly increased (2). It is a great challenge to actively prevent and treat PPH and reduce maternal morbidity and mortality.

Severe PPH is defined as a blood loss of ≥1,000 ml within 24 h after delivery (3). If patients with severe PPH are unresponsiveness to uterine massage and first-line uterotonic agents, further invasive procedures may be required. Conservative surgical procedures include intrauterine balloon tamponade (IUBT), uterine compression sutures, pelvic vessel ligation, and arterial embolization. Hysterectomy is performed when all conservative life-saving treatments have failed to achieve hemostasis, which is associated with high morbidity and loss of fertility (4–8).

IUBT has been used in China since 2012 and is recommended for the treatment of PPH in the guidelines (3, 9). In recent years, IUBT has been widely used as a second-line conservative management for severe PPH because of its relatively non-invasive nature, and the use of IUBT is associated with a reduction in the use of more invasive procedures and has few adverse effects on subsequent menstrual and reproductive function (10–13). However, the application of IUBT is mostly empirical, and evidence for its recommended use is limited. In 2020, a meta-analysis showed that the total success rate of IUBT in the treatment of PPH is 85.9%. Nevertheless, evidence on the efficacy and effectiveness of IUBT from randomized and non-randomized studies has been contradictory (14).

Further study is needed on the application of IUBT to determine the most effective scheme and health delivery strategy. However, prospective randomized controlled trials of PPH management are very difficult to perform, in particular in patients with persistent bleeding. The purpose of this study was to review the application of IUBT in severe PPH to determine the risk factors associated with IUBT failure so as to guide the selection of patients with severe PPH.

## Materials and Methods

### Patients and Study Design

This study was approved by the Peking Union Medical College Hospital Review Board (reference number: JS-1151). The need for written informed consent was waived because of the retrospective nature of the study. Our study was performed in compliance with the Declaration of Helsinki.

This was a retrospective cohort study among women who underwent intrauterine balloon tamponade (IUBT) with severe PPH after delivery from October 1, 2016 until September 30, 2017 in 14 representative hospitals in 10 provinces from four major economic zones in China (including two secondary and 12 tertiary hospitals, seven general hospitals, and seven women and children's hospitals). The data collection methods are described in detail elsewhere (15). In brief, we included all of the deliveries in the hospitals after 20 weeks of gestation to assess complications of delivery. Specially assigned reporting clinicians in each hospital were responsible for collecting and reporting information regarding pregnant women who delivered using a data-collection system. All of the data entered were reviewed and verified by a second person in order to ensure the accuracy of the data entered into the database. We chose eligible cases and exported them from the central database system. In order to protect the privacy of patients, personal identifying information was removed during analysis for all of the cases.

All of the maternity units in the study followed the 2014 guidelines of the Chinese Medical Association of Obstetrics and Gynecology for the prevention and management of PPH. After vaginal delivery or cesarean section, patients with PPH were first managed by uterine massage and first-line uterotonic agents were used (oxytocin, carbetocin, prostaglandin F2α-Hemabate, ergometrine, and misoprostol); if these failed, IUBT was applied according to the clinical setting as decided by the attending senior obstetrician. The balloon tamponade insertion was performed by the attending senior obstetrician followed the manufacturer's recommended instructions. Its intrauterine position was monitored by abdominal ultrasound scanning. The types of IUBT used for PPH were not described in this study, but Bakri balloon (Cook woman's health, USA) was the most commonly used. After the operation, the color, quantity of uterine cavity drainage fluid, height of uterine fundus, and vital signs were closely monitored. Hemoglobin and coagulation function were dynamically monitored. If hemostasis was achieved, the balloon was kept inflated up to 24–48 h. Antibiotics were routinely used after operation to prevent infections. Patients who were given IUBT when the total blood loss was <1,000 ml or had other invasive procedures before IUBT were excluded from this study.

Demographic and baseline clinical data were obtained from the electronic medical records system and included maternal age, gravidity, parity, multiple gestation, pregestational body mass index (BMI), assisted reproductive technique, gynecologic and obstetric history, major medical history, and major family history. The perinatal outcomes included gestational age at birth, birthweight, the method of delivery, and PPH. The PPH outcomes included causes of PPH, estimated blood loss (EBL), transfusion of any blood products (red blood cells, platelets, fresh frozen plasma, or cryoprecipitate), invasive procedures for severe PPH to stop the bleeding (uterine compression sutures, pelvic artery ligation, uterine embolization, or peripartum hysterectomy), and maternal postpartum complications, such as fever, thromboembolic events, or admission to an intensive care unit.

The grading criteria of BMI were based on the recommendation of the Working Group on Obesity in China: underweight: BMI < 18.5 kg/m^2^, normal weight: BMI 18.5–23.9 kg/m^2^, overweight: BMI 24.0–27.9 kg/m^2^, and obesity: BMI ≥ 28.0 kg/m^2^ (16).

PPH is defined as a blood loss of ≥500 ml within 24 h after vaginal delivery or ≥1,000 ml after cesarean delivery. Severe PPH is defined as a blood loss of ≥1,000 ml within 24 h after delivery, unresponsiveness to uterine massage and first-line uterotonic agents, and the need for additional surgical or radiological interventions including hysterectomy (3). Each patient was assigned a single primary cause of PPH. Some patients may have more than one causes of PPH, the main cause of bleeding was assessed by the attending senior obstetrician. Placenta abnormalities were given as the primary cause for women with placenta praevia, placenta accreta spectrum (PAS), or placental abruption. The diagnosis of PAS was made either pathologically or clinically when the placenta did not detach normally from the uterus after delivery of the fetus. “Lacerations” was given as the primary cause for women with lacerations of the cervix, vagina, or perineum. “Uterine atony” was given as the primary cause for women with “global” or “lower uterine segment” atony or “softness.”

The primary outcome was failure of IUBT, which was defined as the need of additional invasive procedures, including conservative surgical procedures (including uterine compression sutures or pelvic vessel ligation), uterine embolization, or peripartum hysterectomy and factors of associated with IUBT failure.

### Statistical Analysis

SPSS (version 25.0, Chicago, IL, USA) was used for Student's *t*-tests, chi-square tests, or Fisher's exact-probability tests. Chi-square tests or Fisher's exact-probability tests were used to compare categorical variables that were presented as counts and percentages between groups with successful and failed IUBT. Student's *t*-test was used to compare continuous variables that are presented as means ± standard deviations. Least absolute shrinkage and selection operator (LASSO) is the most commonly used approach to perform variable selection with regularization. LASSO regression was performed to select the potential risk factors for the prediction of IUBT failure, which were then included in multivariate logistic models. These models were constructed to estimate the risk factors of IUBT failure. Akaike information criterion (AIC) was used to evaluate the quality of the fit of the models. Area under the curve (AUC) used to assess the predictive performance of the selected model. LASSO regression was performed with the “glmnet” package in R (version 4.0.2). Statistical significance was accepted when the *P* < 0.05.

## Results

A total of 99,650 deliveries occurred from October 1, 2016 to September 30, 2017. The frequency of primary PPH was 5.3% (*n* = 5,267), and the frequency of severe PPH was 0.9% (*n* = 863). Among the patients, 106 cases of severe PPH were managed with IUBT, 20 (18.9%) after vaginal delivery and 86 (81.1%) during or after cesarean section. The global success rate was 70.8% (75/106) and was significantly higher after vaginal deliveries (18/20, 90%) than cesareans (57/86, 66.3%, *P* < 0.05).

**Figure 1:**
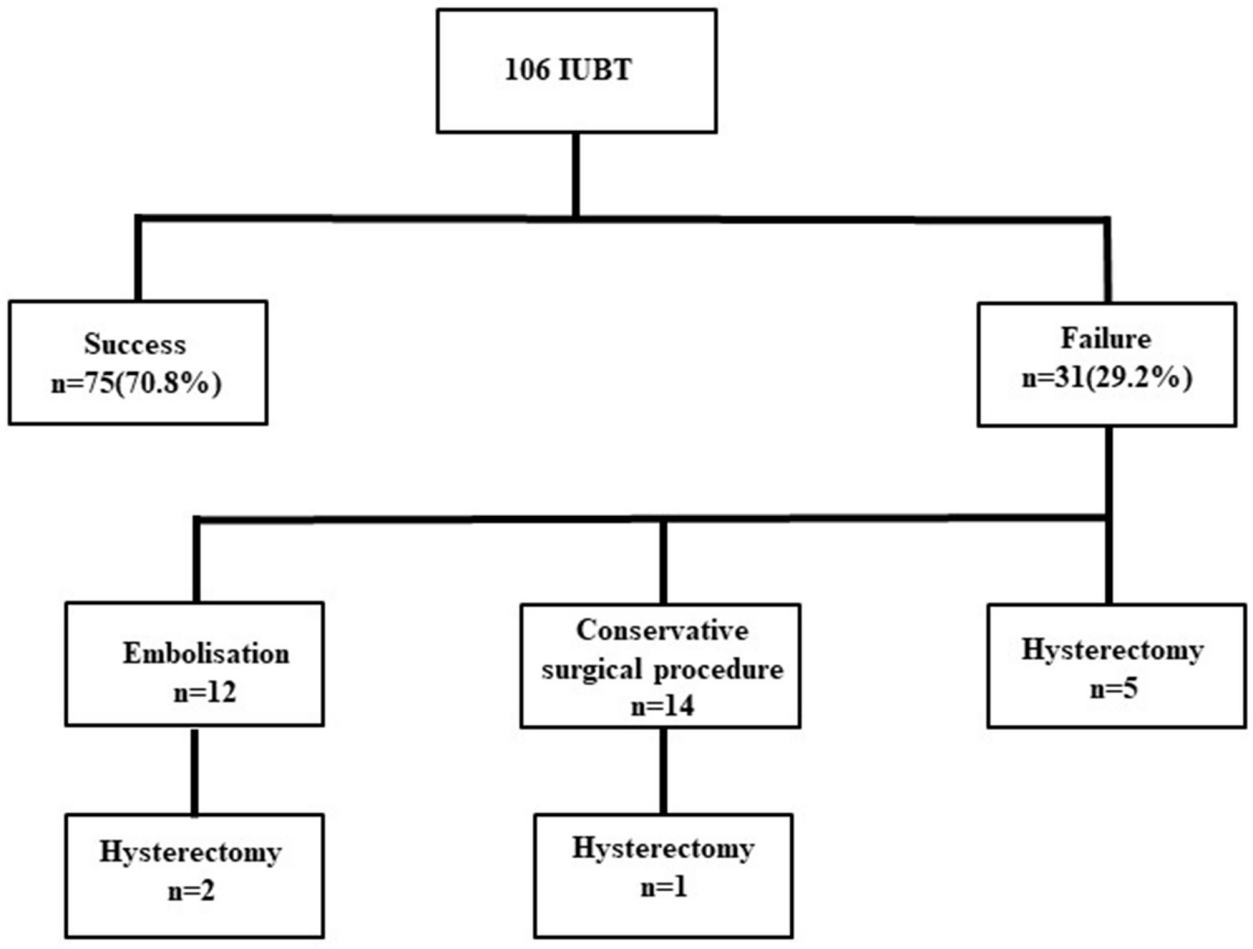
Flowchart. IUBT, intrauterine balloon tamponade.

Among the 31 failures, 12 cases underwent embolization and two cases failed, leading to hysterectomy. Fourteen other cases needed conservative surgical procedures, and one case failed, leading to hysterectomy. Hysterectomy was performed immediately after IUBT failure in five cases. There were no deaths (Figure 1).

General and obstetric characteristics of the women with IUBT are presented in Table 1. In the failure group, compared with the success group, multiparous deliveries were significantly more frequent (71.0 vs. 44.0%, *P* < 0.05), and the frequency of prior cesarean delivery was significantly higher (61.3 vs. 28.0%, *P* < 0.05). The rate of cesarean delivery in the failure group was higher (93.5 vs. 76.0%, *P* < 0.05). Women in the failure group delivered earlier (36.16 ± 2.54 vs. 37.41 ± 2.35 weeks, *P* < 0.05) and had lower birth weights (2778.97 ± 687.29 vs. 3093 ± 671.59 g, *P* < 0.05) in our univariate analysis. There were no differences in other general and obstetric characteristics between the success and failure groups.

**Table 1:** General and obstetric characteristics of the study population.

**Characteristic**	**Success (*n* = 75)**	**Failure (*n* = 31)**	***P*-value**
Maternal age, years^*^	31.40 ± 4.90	32.29 ± 4.13	0.377
<35, *n* (%)	57 (76.0)	21 (67.7)	0.380
≥35, *n* (%)	18 (24.0)	10 (32.3)	
Obesity, *n* (%)	3 (4.0)	2 (6.5)	0.588
Multiparous, *n* (%)	33 (44.0)	22 (71.0)	0.011
Parity, n^*^	1.64 ± 0.77	1.81 ± 0.7	0.299
Preeclampsia, *n* (%)	0 (0.0)	1 (3.2)	0.292
Multiple gestation, *n* (%)	10 (13.3)	5 (16.1)	0.707
Prior cesarean delivery, *n* (%)	21 (28.0)	19 (61.3)	0.001
Known uterine myoma, *n* (%)	2 (2.7)	1 (3.2)	1.000
Assisted reproductive technology, *n* (%)	12 (16.0)	3 (9.7)	0.396
Gestational age at delivery, weeks^*^	37.41 ± 2.35	36.16 ± 2.54	0.017
Mode of delivery, *n* (%)			0.036
Vaginal delivery	18 (24.0)	2 (6.5)	
Cesarean delivery	57 (76.0)	29 (93.5)	
Birthweight, g^*^	3093 ± 671.59	2778.97 ± 687.29	0.032
Birthweight ≥ 4,000 g	5 (6.7)	1 (3.2)	0.486

* *Data are given as means ± standard deviation*.

Table 2 shows the characteristics of severe PPH. The cause of severe PPH in the failure group was mainly due to PAS (17/31, 54.8%) and uterine atony (8/31, 25.8%) in this cohort study. There was a higher frequency of PAS (54.8 vs. 6.7%, *P* < 0.05) in the failure group. In contrast, patients with uterine atony were of lower frequency in the failure group (25.8 vs. 66.7%, *P* < 0.05). There were no significant differences in the proportions of patients with placenta previa and placental abruption between the groups. EBL was significantly higher in the failure group (2746.13 ± 1573.1 vs. 1560.56 ± 688.55 ml, *P* < 0.05).

**Table 2:** PPH characteristics.

**Characteristic**	**Success (*n* = 75)**	**Failure (*n* = 31)**	***P*-value**
Cause of hemorrhage, *n* (%)			
Uterine atony	50 (66.7)	8 (25.8)	0.000
Placenta abnormalities			
Placenta praevia	18 (24.0)	6 (19.4)	0.603
Placenta accreta spectrum	5 (6.7)	17 (54.8)	0.000
Placental abruption	2 (2.7)	0 (0.0)	1.000
Estimated blood loss, ml^*^	1560.56 ± 688.55	2746.13 ± 1573.1	0.000

*PPH, postpartum hemorrhage*.

* *Data are given as means ± standard deviation*.

The frequency of coagulopathy was significantly higher in the failure group (9.7 vs. 1.3%, *P* < 0.05). All coagulopathy developed due to the progression of PPH. The volumes of packed red blood cells and fresh frozen plasma transfusions were significantly higher in the failure group. Patients who underwent intensive care unit admission were more common in the failure group (38.7 vs. 14.7%, *P* < 0.05). No women died in the study (Table 3).

**Table 3:** Management of complications of PPH undergoing intrauterine balloon tamponade.

**Characteristic**	**Success (*n* = 75)**	**Failure (*n* = 31)**	***P*-value**
Units of red blood cells transfused^*^	3.53 ± 3.51	7.61 ± 5.01	0.000
Fresh frozen plasma transfusion, ml^*^	289.6 ± 316.72	824.84 ± 888.15	0.003
Platelets transfusion, U^*^	0.01 ± 0.06	0.1 ± 0.4	0.217
Coagulopathy	1 (1.3)	3 (9.7)	0.040
Admission to intensive care unit, *n* (%)	11 (14.7)	12 (38.7)	0.006
Fever, *n* (%)	1 (1.3)	2 (6.5)	0.204
Thromboembolic event, *n* (%)	1 (1.3)	1 (3.2)	0.501
Maternal death, *n* (%)	0 (0.0)	0 (0.0)	-

*PPH, postpartum hemorrhage*.

* *Data are given as means ± standard deviation*.

**Figure 2:**
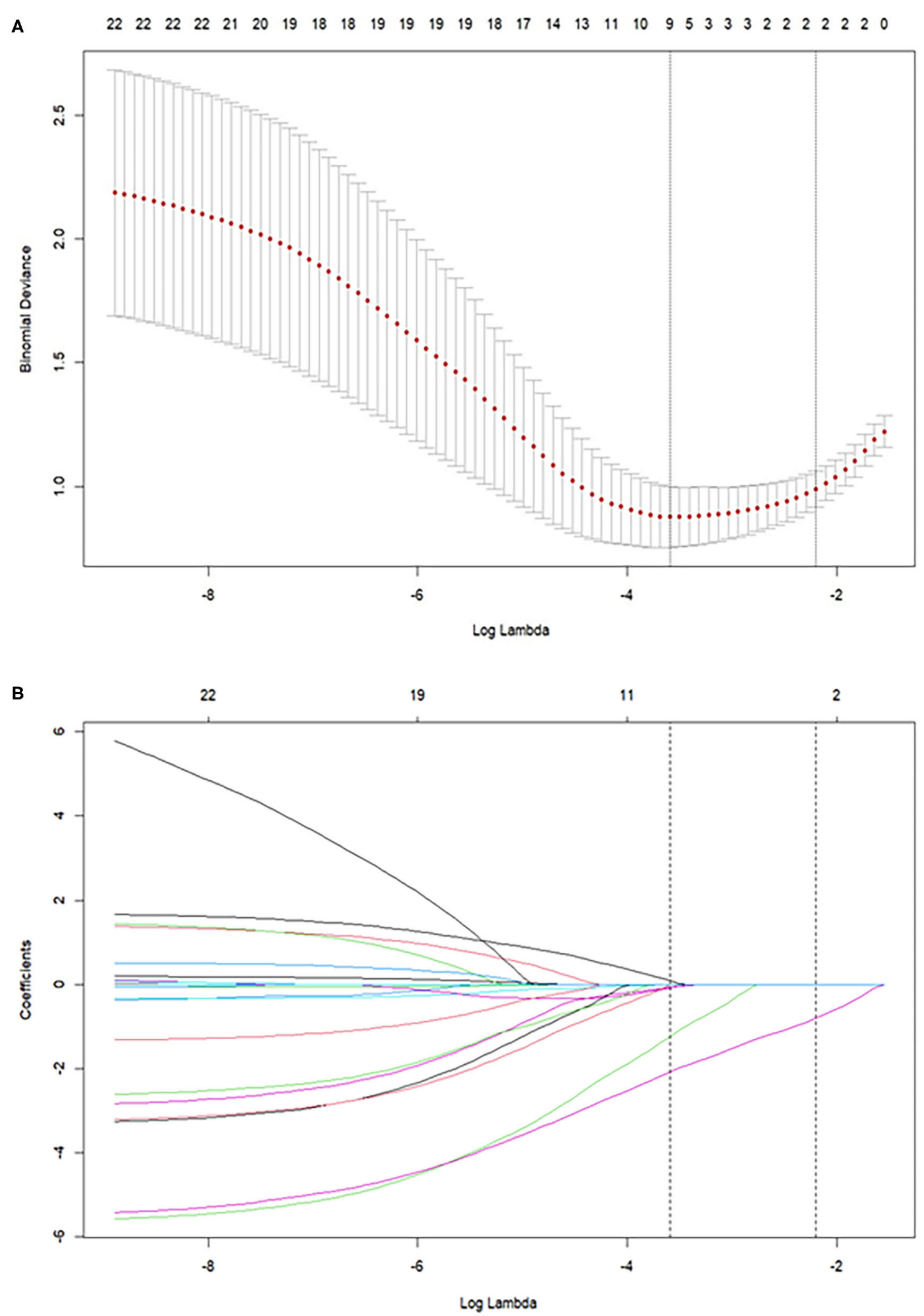
**(A)** Binomial Deviance of the LASSO model with different log (lambda). **(B)** LASSO coefficient profiles of the risk factors predicting IUBT failure. The best combination of risk factors was selected by LASSO logistic regression analyses, with risk factors selected by the log (lambda) at which the minimal Binomial Deviance was achieved. LASSO, least absolute shrinkage and selection operator; IUBT, intrauterine balloon tamponade.

The list of the selected risk factors predicting IUBT failure in LASSO regression is given in Tables 1, 2. The results showed that obesity, multiple gestation, cesarean delivery, EBL, and PAS had predictive significance for IUBT failure (Figure 2). The associated risk factors were included in the logistic model. Ultimately, these models identified multiple gestation [odds ratio (OR) 15.52; 95% confidence interval (CI) 2.27–150.454], EBL (OR 1.166; 95% CI 1.087–1.274), and PAS (OR 26.993; 95% CI 5.815–205.528) as independent risk factors for IUBT failure (Table 4). The AUC is 0.89.

**Table 4:** Multivariate regression analysis for the outcome of intrauterine balloon tamponade failure.

**Variable**	***P***	**OR**	**95% CI**
Obesity	0.534	1.063	0.880–1.301
Multiple gestation	0.009	15.52	2.27–150.454
Estimated blood loss	<0.001	1.166	1.087–1.274
Cesarean delivery	0.710	1.438	0.236–12.808
Placenta accreta spectrum	<0.001	26.993	5.815–205.528

*OR, odds ratio; CI, confidence interval*.

## Discussion

### Main Findings

This retrospective cohort study found that the global success rate of IUBT in the management of severe PPH was 70.8% (75/106). We demonstrated that multiple gestation, EBL, and PAS were risk factors for IUBT failure.

### Interpretation

The success rate of IUBT alone in the management of severe PPH was 70.8%, which was slightly lower than in previous studies (8, 11, 17). This may be related to the higher proportion of PAS and cesarean sections. Our study showed that the IUBT success rate for PAS was lowest among the causes of PPH. In addition, the IUBT success rate was lower for cesarean deliveries than for vaginal deliveries.

We found that PAS was associated with IUBT failure. Previous studies have demonstrated that placenta accreta is negatively associated with the success of IUBT (11, 18). Pala et al. (19) showed that in cases with less severe invasion and a smaller PAS area, the success rate of Bakri balloons was 84.2%. Our study indicates that IUBT is not as effective for the management of severe PPH in patients with severe PAS in view of the high failure rate. There were 22 cases with diagnosis of PAS in the present study, and of the 17 patients with balloon failure, six cases who underwent cesarean hysterectomy had confirmed placenta increta and percreta. In cases of PAS disorders, the placenta was separating from the uterus to avoid leaving retained placental tissues in the uterine cavity, often resulting in massive obstetric hemorrhage and requiring hysterectomy. Surgical risks increased with the depth of placental invasion. Whether a balloon tamponade should be recommended in cases of PAS requires further investigation. It should be noted that IUBT is easy to deploy and the balloon can be inserted quickly. Even in cases with failure the balloon may provide a temporary tamponade effect and enough time to prepare for other interventions or patient transfer.

Our study showed that EBL was associated with IUBT failure. All of the cases involved severe PPH in this study. Women with persistent postpartum hemorrhage may develop coagulopathy with blood loss, dilution, and consumption of platelets and coagulation factors, requiring high rates of blood transfusions. Previous studies have shown that EBL before IUBT is associated with IUBT failure (8, 11, 20), with an odds ratio for EBL before IUBT (>1,500 ml) of 3.2 (8). Howard and Grobman (21) reported that women receiving IUBT at higher EBL quartiles had more frequent packed red blood cell transfusions and more hysterectomies. IUBT needs to be used earlier in the management of persistent PPH. It is necessary to judge the bleeding characteristics and intervention timing as soon as possible, which influences the success rate of invasive procedures and impacts the prognosis of women with PPH.

Multiple gestation is well-known to be highly associated with PPH (22, 23), which was also associated with IUBT failure in our study. In our study, multiple pregnancies had higher rates of cesarean deliveries (14/15, 93.3%). Moreover, multiple gestations are at risk for uterine atony due to overdistension. It would seem reasonable to conclude that multiple gestations are at greater risk for IUBT failure. In addition, as previously mentioned, multiparous and prior cesarean delivery were also significantly increased in the failure group in our study. These outcomes reiterate the potential severity of PPH associated with these high-risk factors.

China's universal two-child policy announced in October 2015 has been associated with a rise in births in China and with changes in health-related birth characteristics. Between July 2016 and December 2017, women giving birth have been more likely to be multiparous, aged 35 and over, and the number of cesarean deliveries has risen for women who have had prior cesarean sections (2). Repeat cesarean deliveries further lead to a corresponding increase in the incidence of placenta previa and PAS (24). In 2018, national maternal monitoring found that the causes of PPH due to placental factors account for 60.49% of cases. Thus, we should increase the early identification of women at high risk of PPH. Pregnancies at high risk for PPH should be transferred to a referral center for complicated pregnancies as soon as possible, and other modalities of management should be anticipated early so as to provide higher quality perinatal health care and reduce severe maternal morbidity and mortality.

In our study, the success rates of IUBT caused by uterine atony and previa were, respectively, 86.2 and 75%, which is consistent with previous studies (17, 25, 26). Therefore, we think that IUBT should be attempted for these two causes of PPH.

### Strengths and Limitations

Our study was a multicenter retrospective cohort study to assess the success rate of IUBT in treating severe PPH, and all of the units that managed PPH followed the 2014 guidelines of the Chinese Medical Association of Obstetrics and Gynecology. The main limitation of the present study is its retrospective nature, which may raise some concerns regarding data quality and the risk of selection and information bias. Second, some patients may have more than one cause of PPH, and uterine atony may be secondary to placental abnormalities and overlap each other, so it is difficult to identify the primary cause of PPH. Third, the EBL before IUBT and the interval time between the diagnosis of PPH and balloon insertion were not recorded, so we could not further evaluate when to use the IUBT. Last, the diagnosis of PPH can be very subjective because it is impossible to accurately measure the amount of bleeding. This will always be a limitation in PPH research.

## Conclusion

In conclusion, we demonstrated that IUBT is effective when used before any invasive procedures in severe PPH. Our data demonstrated factors affecting the failure of IUBT, including multiple gestation, EBL, and PAS. The presence of factors affecting IUBT failure should be recognized early, and other modalities of management should be anticipated early on.

## Data Availability Statement

The raw data supporting the conclusions of this article will be made available by the authors, without undue reservation.

## Author Contributions

JG, JL, and CL designed this study and analyzed and interpreted the data. JG, JL, XW, JH, JS, XL, and SL provided study materials or patients. CL collected data and wrote the manuscript. All authors discussed the results, contributed to the article, and approved the final manuscript.

## Conflict of Interest

The authors declare that the research was conducted in the absence of any commercial or financial relationships that could be construed as a potential conflict of interest.

## Abbreviations

BMI: body mass index
CI: confidence interval
EBL: estimated blood loss
IUBT: intrauterine balloon tamponade
LASSO: least absolute shrinkage and selection operator
OR: odds ratio
PAS: placenta accreta spectrum
PPH: postpartum hemorrhage.
